# World Stroke Organization (WSO): Global intracerebral hemorrhage factsheet 2025

**DOI:** 10.1177/17474930241307876

**Published:** 2025-01-06

**Authors:** Adrian R Parry-Jones, Rita Krishnamurthi, Wendy C Ziai, Ashkan Shoamanesh, Simiao Wu, Sheila O Martins, Craig S Anderson

**Affiliations:** 1 Geoffrey Jefferson Brain Research Centre, Manchester Academic Health Science Centre, Northern Care Alliance and University of Manchester, Manchester, UK; 2 Division of Cardiovascular Sciences, Faculty of Biology, Medicine and Health, University of Manchester, Manchester, UK; 3 National Institute of Stroke and Applied Neurosciences, School of Clinical Sciences, Auckland University of Technology, Auckland, New Zealand; 4 Departments of Neurology, Neurosurgery and Anaesthesiology/Critical Care Medicine, The Johns Hopkins University School of Medicine, Baltimore, MD, USA; 5 Department of Medicine (Neurology), McMaster University and Population Health Research Institute, Hamilton, ON, Canada; 6 Department of Neurology, West China Hospital, Sichuan University, Chengdu, P.R. China; 7 Centre for Cerebrovascular Diseases, West China Hospital, Sichuan University, Chengdu, P.R. China; 8 Neurology Service, Hospital de Clínicas de Porto Alegre, Universidade Federal do Rio Grande do Sul, Porto Alegre, Brazil; 9 Neurology Service, Hospital Moinhos de Vento, Porto Alegre, Brazil; 10 Institute of Science and Technology for Brain-Inspired Intelligence, Fudan University, Shanghai, China; 11 The George Institute for Global Health, Sydney, NSW, Australia

**Keywords:** Intracerebral hemorrhage, epidemiology, risk factors

## Abstract

**Background:**

Intracerebral hemorrhage (ICH) is stroke caused by non-traumatic bleeding into the brain.

**Aim:**

This factsheet provides summary statistics for ICH from the 2021 Global of Burden of Diseases Study.

**Methods:**

Data were downloaded from the GBD results platform using “intracerebral hemorrhage” as a Level 4 cause of death or injury, extracting key metrics (number, percent, rate) for measures (incidence, disabilty adjusted life years [DALYs], deaths) described in this factsheet.

**Results:**

Globally, stroke was the third leading cause of death in 2021, and ICH accounted for 28.8% of incident strokes. There were estimated to be 7,252,678 deaths due to stroke in 2021 of which ICH accounted for 3,308,367 (45.6%). When considering the burden of ICH in terms of DALYs, ICH accounts for nearly half of the burden of stroke at 49.5%, compared to 43.8% caused by ischemic stroke. ICH must therefore be considered on an equal footing with ischemic stroke, so that efforts can be made to reduce its burden through public health, research, and healthcare provision. Although the overall age-standardized incidence of ICH has been decreasing since 1990, the rate of reduction has been much slower in regions with lower socio-demographic index (SDI). Most of the burden of ICH lies in areas with lower SDI, with 94.2% of DALYs lost to ICH outside areas of high SDI. Geographically, the majority of DALYs due to ICH occur in Southeast Asia, East Asia, and Oceania, with 53.3% of global DALYs lost in these regions alone. The risk factors for ICH are dominated by high systolic blood pressure, which accounts for at least 50% of the burden of ICH, regardless of SDI. Areas with middle or high-middle SDI have a greater proportion of the burden of ICH accounted for by ambient particulate pollution, smoking, and diets high in sodium, whereas household air pollution from solid fuels accounts for much more of the risk of ICH in low SDI regions.

**Conclusion:**

This World Stroke Organization (WSO) Global ICH Fact Sheet 2025 provides the most updated information on ICH that can be used to support communication with all internal and external stakeholders, inform healthcare policy, and raise public awareness. All statistics have been reviewed and approved for use by the WSO Executive Committee.

## Overview

Stroke was the third leading cause of death in the 2021 Global Burden of Disease (GBD) study,^1^ having been displaced from the second leading cause of death in 2019 by COVID-19.^2^ Intracerebral hemorrhage (ICH) is stroke caused by non-traumatic hemorrhage into the brain.^3^ Together with ischemic stroke and subarachnoid hemorrhage, it is one of three major pathological types of stroke. The most recent 2021 Global Burden of Disease data show that globally stroke has an annual, age-standardized incidence of 141.55 (95% uncertainty interval [UI] 127.97 to 155.81) per 100,000 population. ICH has an incidence of 40.83 (95% UI 36.2 to 45.21) per 100,000, accounting for 28.8% of incident strokes. Ischemic stroke has over twice the incidence (92.39 [95% UI 79.84 to 105.82] per 100,000), accounting for 65.3% of incident strokes. However, when considering the overall burden of ICH in relation to ischemic stroke, ICH accounts for nearly as many deaths and more disability adjusted life years (DALYs). There were estimated to be 7,252,678 deaths in 2021 due to stroke of which ICH accounted for 3,308,367 (45.6%), compared to 3,591,499 (49.5%) for ischemic stroke. Stroke caused 160,457,220 DALYs, with ICH accounting for 49.6% of total DALYs due to stroke, and ischemic stroke less, at 43.8%. Death and disability after ICH can be reduced by consistent and effective delivery of guideline-based care, including blood pressure management, neurosurgery, and high-quality supportive care on dedicated stroke units and critical care units.^4^ Combining interventions in acute care bundles has shown benefit in both high- and low-middle income countries.^5,6^

**Figure 1.**
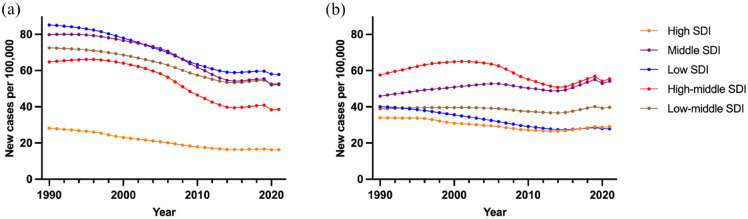
Age-standardized (a) and absolute (b) incidence of intracerebral hemorrhage, divided by socio-demographic index (SDI) from 1990 to 2021.

Annual GBD data from 1990 to 2021 shows that the age-standardized incidence of ICH is considerably lower in high socio-demographic index (SDI) countries but is decreasing across all SDI groups, although the trends are slower in low- and low-middle SDI countries (Figure 1(a)). Absolute incidence shows a more complex pattern due to changing age distributions. Following a gradual reduction in absolute incidence for all SDI levels from approximately 2005 to 2014, a consistent increase in incidence across all SDI levels has occurred since, except for low SDI countries (Figure 1(b)).

**Figure 2.**
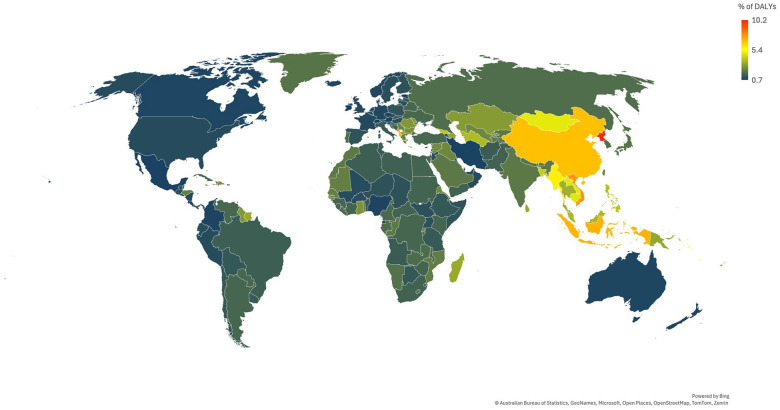
Percent of total DALYs lost to intracerebral hemorrhage by country in 2021.

**Figure 3.**
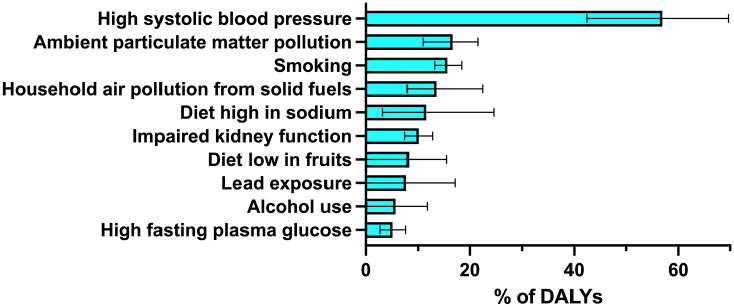
Percentage of all DALYs caused by intracerebral hemorrhage, attributable to specific risk factors. The top 10 risk factors are shown with 95% uncertainty interval. Data from Global Burden of Disease study, all ages, both sexes, globally, in 2021.

Most of the burden of ICH occurs in areas with lower SDI, with 94.2% of DALYs lost to ICH occurring outside areas of high SDI. Geographically, the majority of DALYs are lost in Southeast Asia, East Asia, and Oceania, with 53.3% of global DALYs lost in this region alone (Figure 2). ICH is increasingly common with age (Table 1), with the highest incidence in those aged 70 years or more. The incidence, prevalence, deaths, and DALYs are all slightly higher in men than women. The risk factors accounting for ICH are dominated by high systolic blood pressure (Figure 3). It remains the leading risk factor for ICH regardless of SDI status, contributing to 51.8% of the ICH burden in low SDI areas compared to 58.7% of the ICH burden in high-middle SDI areas. There is considerably more variation in the importance of ambient particulate matter pollution by SDI, accounting for 20.3% of the burden in middle SDI and 20.2% in high-middle SDI areas versus 7.5% in low SDI and 11.5% in high SDI areas. A similar pattern is noted for smoking, with the greatest contribution in middle and high-middle SDI regions (17.5% and 19.9%, respectively), compared to low SDI regions (7.4%). Likewise, a diet high in sodium accounts for more risk in high-middle (15.5%) and middle (14.1%) SDI regions versus low SDI (4.7%) regions. These risks may thus represent an important focus for regions with middle or high-middle SDI, modifiable through public health interventions. For low SDI regions, household air pollution from solid fuels accounts for much more of the risk at 37.0%, compared to 0.04% in high SDI and 8.0% in middle SDI regions.

**Table 1.** GBD 2021 estimates for global incidence, prevalence, mortality, and disability-adjusted life years (DALYs) for intracerebral hemorrhage.

Metric	Number	Crude rate per 100,000 per year (95% UI^a^)	Age-adjusted rate per 100,000 per year (95% UI)	Approved statement for use in WSO materials
Incidence				
Ages (all), sexes (both)	3,444,338	43.65 (38.69-48.31)	40.83 (36.2-45.21)	There are over 3.4 million new cases of intracerebral hemorrhage each year. Globally, 29% of all incident strokes are due to intracerebral hemorrhage
15-49 years	613,694	15.54 (12.66-18.86)	–	Each year, 18% of all intracerebral hemorrhages occur in people aged between 15 and 49 years
<70 years	1,943,503	26.27 (22.25-30.23)	–	Each year, 56% of all intracerebral hemorrhages occur in people under the age of 70 years
Men (all ages)	1,920,625	48.51 (42.78-53.92)	48.71 (43.07-54.27)	Each year, 56% of all intracerebral hemorrhages occur in men
Women (all ages)	1,523,713	38.75 (33.88-43.04)	33.61 (29.47-37.22)	Each year, 44% of all intracerebral hemorrhages occur in women
Prevalence				
Ages (all), sexes (both)	16,603,836	210.41 (192.1-230.42)	194.51 (177.99-212.53)	Globally, there are over 16 million people currently living who have experienced an intracerebral hemorrhage
15-49 years	5,578,619	141.28 (127.88-158.35)	–	34% of people who have experienced an intracerebral hemorrhage and are currently living are people aged between 15 and 49 years
<70 years	13,567,587	183.42 (167.18-201.27)	–	82% of people who have experienced an intracerebral hemorrhage and are currently living are aged less than 70 years
Men (all ages)	9,349,225	236.13 (214.55-258.66)	225.08 (204.86-245.59)	56% of people who have experienced an intracerebral hemorrhage and are currently living are men
Women (all ages)	7,254,612	184.50 (169.32-201.55)	166.10 (152.2-181.16)	44% of people who have experienced an intracerebral hemorrhage and are currently living are women
Deaths				
Ages (all), sexes (both)	3,308,367	41.92 (38.28-45.55)	39.09 (35.65-42.45)	Over three million people die from intracerebral hemorrhage annually
15-49 years	263,710	6.68 (6.09-7.35)	–	About 8% of all deaths from intracerebral hemorrhage occur in people 15–49 years old
<70 years	1,524,337	20.61 (18.86-22.33)	–	46% of deaths from intracerebral hemorrhage occur in people under 70 years old
Men (all ages)	1,822,740	46.04 (41.18-51.72)	47.43 (42.33-53.18)	55% of all deaths from intracerebral hemorrhage are in men
Women (all ages)	1,485,627	37.78 (33.33-42.41)	32.09 (28.31-35.99)	45% of all deaths from intracerebral hemorrhage are in women
DALYs				
Ages (all), sexes (both)	79,457,427	1,006.89 (921.88-1083.21)	923.64 (844.83-993.18)	Almost 79 million years of healthy life is lost each year due to intracerebral hemorrhage-related death and disability
15-49 years	13,794,941	349.36 (318.36-381.99)	–	17% of healthy life lost due to intracerebral hemorrhage-related death and disability affects people between that age of 15 and 49 years old
<70 years	52,995,320	716.44 (659.73-775.55)	–	67% of healthy life lost due to intracerebral hemorrhage-related death and disability affects people under the age of 70 years
Men (all ages)	45,786,602	1156.41 (1042.85-1290.26)	1123.09 (1014.11-1251.66)	Men account for 58% of healthy life lost due to intracerebral hemorrhage-related disability
Women (all ages)	33,670,825	856.34 (759-950.46)	742.51 (657.65-822.69)	Women account for 42% of healthy life lost due to intracerebral hemorrhage-related disability

a 95% uncertainty interval (UI) represents a range of values that reflects the certainty of an estimate. In GBD, every estimate is calculated 1000 times, each time sampling from distributions rather than point estimates for data inputs, data transformations, and model choice.

The vision of the World Stroke Organization (WSO) is for a world where people live free from the effects of stroke. This vision drives a global effort to improve stroke prevention, treatment, rehabilitation, and support.^7^ The WSO advocates with governments, system leaders, healthcare providers, and the general public, to drive improvements in public health to reducing the risk of stroke, and for the ready availability of acute stroke treatments and rehabilitation for those affected by stroke. Accurate, up-to-date information on the burden of stroke and its subtypes underpin these efforts. ICH has different but overlapping risk factors to ischemic stroke and increasing different treatments, thus it remains important to consider it both as part of the clinical syndrome of stroke and as a separate disease entity.

## Methods

Data in this factsheet have been obtained from the Global Burden of Disease Collaborative Network, Global Burden of Disease Study 2021 Results, Seattle, United States: Institute for Health Metrics and Evaluation (IHME). Data are available from https://vizhub.healthdata.org/gbd-results/. Data were downloaded from the GBD results platform using “intracerebral hemorrhage” as a Level 4 cause of death or injury, extracting key metrics (number, percent, rate) for measures (incidence, DALYs, deaths) described in this factsheet. DALYs by country were extracted for Figure 2. Estimates for risk factors up to level 4 were also extracted for intracerebral hemorrhage. All data are age-standardized, unless stated otherwise.

## Discussion

**Figure 4.**
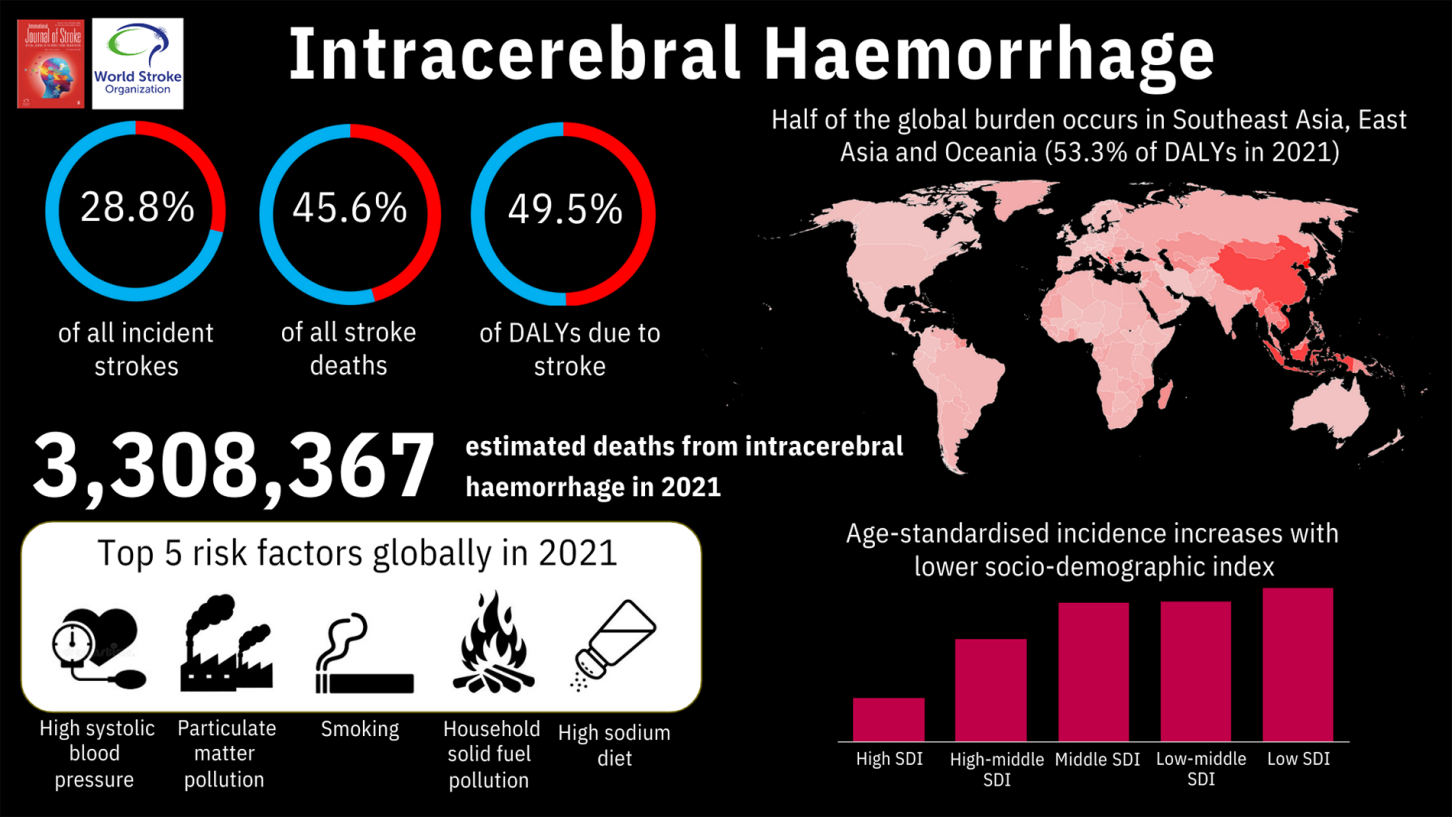
Infographic.

This WSO Global ICH Fact Sheet 2025 and accompanying infographic (Figure 4) provide the most updated information that can be used to inform communication with all internal and external stakeholders, inform healthcare policy, and raise public awareness; all statistics have been reviewed and approved for use by the WSO Executive Committee. The facts are endorsed by the WSO and will be updated every 1–2 years as new data emerges.
